# Comparing the needs and utilization of health services between urban residents and rural-to-urban migrants in China from 2012 to 2016

**DOI:** 10.1186/s12913-018-3522-y

**Published:** 2018-09-17

**Authors:** Lingling Zheng, Ruwei Hu, Zichuan Dong, Yuantao Hao

**Affiliations:** 1 Department of Medical Statistics and Epidemiology, School of Public Health, Sun Yat-sen University, Guangzhou, 510080 Guangdong Province China; 2 Department of health management, School of Public Health, Sun Yat-sen University, Guangzhou, 510080 Guangdong Province China; 3 State Key Laboratory of Infectious Disease Prevention and Control National Institute for Communicable Disease Control and Prevention, Chinese Center for Disease Control and Prevention, No.155, Changbai Road, Changping District, Beijing, 102206 China; 4 Department of Medical Statistics and Epidemiology, School of Public Health, Key Laboratory of Health Informatics, Sun Yat-sen Global Health Institute, Sun Yat-sen University, 74 Zhongshan RoadII, Guangzhou, 510080 Guangdong Province China

**Keywords:** Needs and utilization of health services, Rural-to-urban migrants, Urban residents, China

## Abstract

**Background:**

With a large population of internal migrants from all over the world, China has the largest number of internal floating migrants, and most of them (up to 169 million in 2016) are rural-to-urban migrants. Those migrants have difficulty accessing essential health care services because of Hukou, leading to disparities in health needs and utilization between rural-to-urban migrants and residents. To compare the needs and utilization of health services between urban residents and rural-to-urban migrants in China from 2012 to 2016.

**Method:**

We used longitudinal data from the Chinese Labor Dynamic Survey (CLDS) with three waves in 2012, 2014 and 2016. Descriptive analysis was employed to show self-reported illnesses and health services utilization among locals and migrants in the most recent 2 weeks in China. Chi-square tests and log binomial regression models were constructed to explore factors influencing health care needs and utilization.

**Result:**

A total of 19.97% of respondents were rural-to-urban migrants, with an upward trend from 2012 to 2016. Rural-to-urban migrants (11.99%) had higher needs for health services than urban residents (10.47%) in general, while urban residents and migrants had no differences in needs in 2012. Besides, there was no difference in the utilization of health services between residents and migrants in 2012, 2014 or 2016. In addition, increased age, male sex, poor medical insurance coverage and dissatisfaction with income were found to have negative effects on health care needs.

**Conclusion:**

This study has shown that the rural-to-urban migrants had higher health care needs but the same health care utilization compared with urban residents in China. Health policies focusing on equitable health outcomes should pay more attention to rural-to-urban migrants in China’s health care system reform.

## Background

An estimated 740 million internal migrants globally are on the move^1^. Protection of the human rights regarding the health of migrants has been increasingly recognized and has attracted international attention [1]. WHO has been promoting the health of migrants and committed to adequately address health needs for migrants as part of the global compact for orderly and regular migration.^1^ Migrants face many obstacles in accessing essential health care services due to factors such as irregular immigration status, language barriers, a lack of migrant-inclusive health policies and inaccessible public services. A framework for migrants’ health by the WHO has recognized an urgent need for the health sector to address the impact of migration health more effectively.

China was reported as a country with the largest internal floating population (up to 253 million in 2012) around the world [2]. The primary part of this internal floating population are the rural-to-urban migrants, who always have been called “Peasant workers”.^2^ There were 169 million rural-to-urban migrants in China in 2016 [3]. Internal rural-to-urban migration was modeled as a response to the disparities between the urban and rural sectors [4]. Due to their comparatively poorer social and economic status, these migrants usually lived in poorer environments [1] and had limited access to a range of public services [5]. Moreover, the rapid growth of available wealth and labor welfare has not been distributed evenly among the workforce population, leading to increased disparities in health services utilization among the different sectors of the population [6].

Rural-to-urban migrants do not qualify for public health services and other assistance services [7] such as employment, education, housing and social services [8]. Some researchers have confirmed that rural-to-urban migrants have a higher risk of three main diseases in China: infectious diseases, maternal health and occupational diseases, and injuries [7]. According to the framework for migrants, they have the fundamental right of equality, as all human beings, to the enjoyment of an attainable standard of health, without the distinction of social or economic conditions.

The WHO acknowledges that laws, regulations and policies governing access to health services by migrants vary across countries and are determined by national laws, policies and priorities.^3^ China’s unique Hukou (the nationwide household registration system) originally was designed to regulate rural outflows and serve as a basis for resource allocation to specific groups. As rapid urbanization has occurred over the past 30 years, Hukou no longer limits to urban migration but still affects migrants’ lives in urban areas, such as children’s education, social welfare or access to health care [9], which hinders the implementation of universal health coverage in China. Similarly, the internal passport system may obstruct equity appearing in other countries, including Australia, Japan and some European countries [10]. Experiences from other countries suggest that a multisector approach is required to overcome these obstacles, which must be tailored to address the specific needs of migrants.

Previous studies have focused on immigrants’ health demands in different countries or regions, such as the United States [11], England [12, 13], Australia [14], and Canada [15], as well as different subgroups of a population, such as pregnant women [16], adolescents with mental disorders [17, 18], and HIV-infected adults [19]. In China, more attention is paid to the equity issues of the utilization of health services [20–23]. Most of the previous studies mainly explored the situation or influencing factors of need and utilization of health services among rural-to-urban migrants and showed inequalities in health demands and health services utilization among migrants [24, 25] There is a shortage of research on the discussion of the disparities in health needs and utilization between rural-to-urban migrants and urban residents in China. Therefore, this paper mainly discusses the disparities and utility of health services between rural-to-urban migrants and urban residents by using a nationwide investigative public database and explores some relevant factors governing migrants’ health needs and health services utilization.

## Methods

### Data and sampling

The data^4^ were derived from the Chinese Labor Dynamic Survey (CLDS), which was a nationally representative panel survey of China. CLDS covered 29 provinces, excluding Hong Kong, Macau, Taiwan, Tibet and Hainan, with a multistage cluster, and stratified, probability proportionate to size sampling (PPS) sampling strategy conducted by Sun Yat-sen University. Three waves of follow-up surveys in 2012, 2014 and 2016 have been completed. A total of 22,720 respondents were recruited from urban areas in these three surveys.

### Subjects

The definition by the Chinese government of the floating population [26] refers to population separating from their household register, excluding living in other district/town in the city. In this study, we mainly explored urban residents (who have a nonagricultural household in the urban areas and live in the same urban areas) and rural-to-urban migrants (who have an agricultural household and live in the urban areas).

In 2012, the respondent was identified as belonging to the floating population if they answered ‘no’ to I1.15: “Is your household registration in this city/county?” and ‘yes’ to I1.15.1: “Has it been more than half a year since you were away from your place of household registration?” In 2014 and 2016, the respondent was classified as part of the ‘floating population’ if they chose the answer “in other subdistrict/town of the same county” or “in other county” to I1.3.2: “where is your household registration?” and ‘yes’ to I1.16 (same with I1.15.1 in 2012).

**Fig. 1:**
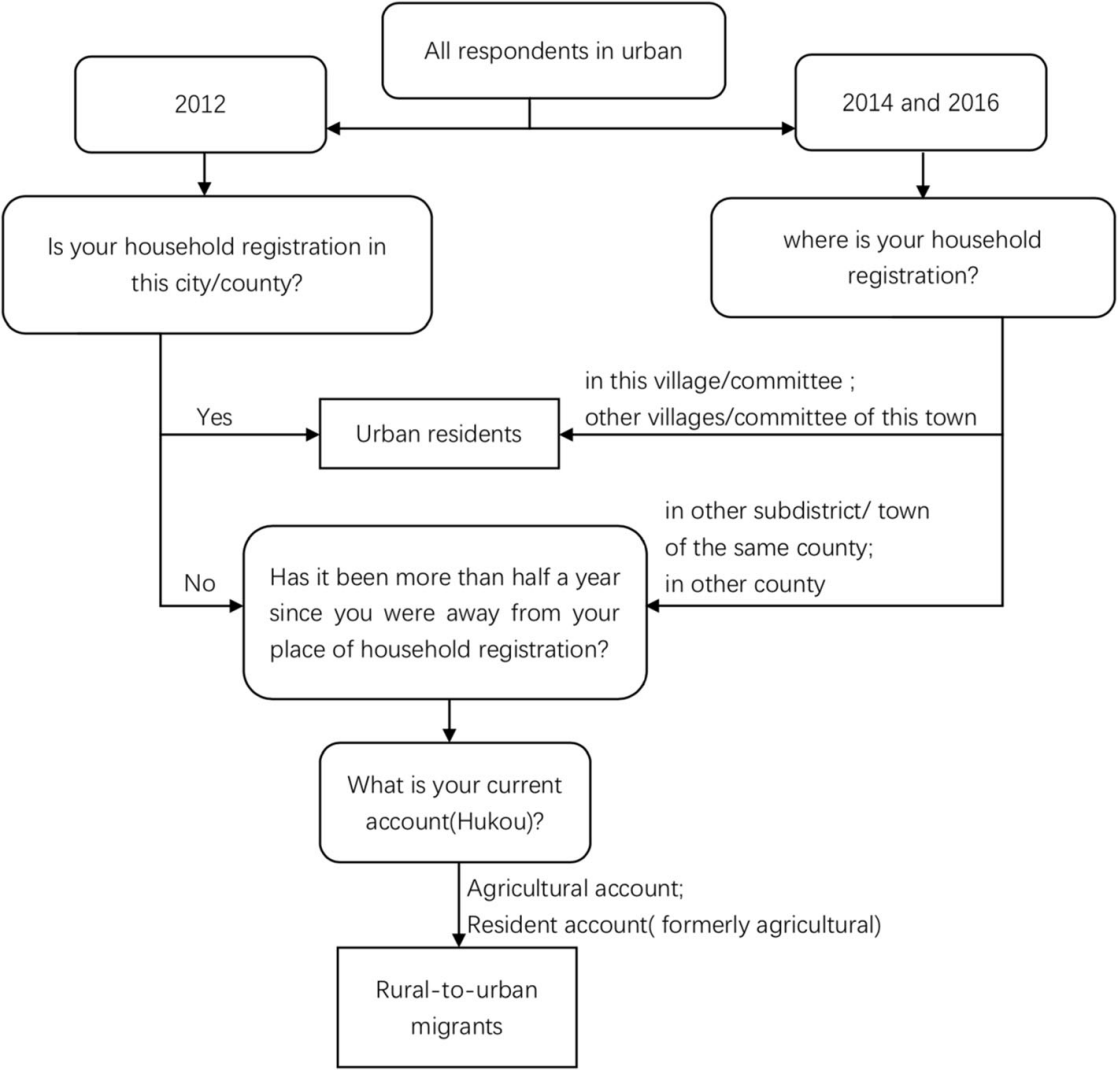
The flow chart of the study subjects

According to the items mentioned above, we could distinguish between the floating population and nonfloating population. Then, according to the type of household, we could further classify them into urban residents and rural-to-urban migrants, with a total of 14,590 respondents in the three surveys. The Fig. 1 showed the flow chart of this study subjects. Moreover, according to the judgment of reliability of the survey by the investigators, the respondents who were considered unreliable and very unreliable were excluded. Finally, 14,119 (96.77%) respondents were included in our analysis.

### Variables and measurement

#### The needs and utilization of health services

The needs of health services were represented by ‘the person being sick in the past two weeks’ (I19.7: “Have you ever been ill over the past two weeks?”)

The utilization of health services was represented by ‘the person visiting the clinic in the past two weeks’ (I19.7.1: “Did you see the doctor over the past two weeks?”)

#### Demographic factors

Demographic characteristics in this paper include age (age 15–24 = 0, age 25–34 = 1, age 35–44 = 2, age 45–59 = 3, age ≥ 60 = 4), gender (female = 0, male = 1), marital status (unmarried = 0, married or living together = 1, divorce or widowed = 2).

#### Socioeconomic factors

Socioeconomic variables include education level (primary or less = 0, junior high school or senior high school = 1, college or above = 2), having medical insurance (yes = 1, no = 0), being employed (yes = 1, no = 0), work time per week and individual income in the past year (analyzed as a continuous variable), self-perception on current job’s income (a 5-point Likert response scale of “very satisfactory”, “satisfactory”, “fairy”, “unsatisfactory”, “very unsatisfactory”), and self-assessment of own social status (point 1–10; the highest social strata = 10, the lowest social strata = 1).

#### Health status

Health status used self-reported health (categorized into 3 groups: “Healthy”, “Fair” and “Unhealthy”.

### Statistical analysis

We used the “mean replacement method” or “Maximum likelihood replacement method” to address the missing continuous or discrete variables data [27–29]. The chi-square test was used to analyze the differences between urban residents and rural-to-urban migrants regarding demographic, socioeconomic, health status and health-related behavior factors. A logistic regression was applied with weighted data to assess influences on the needs and utilization of health services. The forward-stepwise method was performed for variable selection. The statistical significance level was defined as 0.05. All statistical cleaning and analysis were performed using Stata version 13.1.

## Results

### Descriptive analysis

The frequency of urban residents and rural-to-urban migrants was reported by years in Table 1. In general, 19.97% of respondents were rural-to-urban migrants. The proportion of the rural-to-urban migrants shows an upward trend from 2012 to 2016.

**Table 1:** The frequency of urban residents and rural-to-urban migrants by years

	Urban residents		Rural-to-urban migrants		Total (n)
	n	%	n	%	
2012	3650	83.72	710	16.28	4453
2014	4206	79.73	1040	20.27	5523
2016	3444	76.81	2819	23.19	4604
total	11,300	80.03	2819	19.97	14,119

The distribution of demographic, socioeconomic, health status and health-related behavior factors of study respondents were shown in Table 2. Urban residents and rural-to-urban migrants have no difference in gender distribution. Rural-to-urban migrants (36.71 ± 12.18, age 15–34 accounting for 26.67%) had a larger young and single population than urban residents (43.7 ± 13.75, age 15–34 accounting for 48.35%) and the proportion of rural-to-urban migrants (19.65%) who had not been married was higher than that of urban residents (16.89%). Urban residents had higher levels of education than rural-to-urban migrants, and the proportion of urban residents’ education (9.73%) through primary school or lower is lower than that of rural-to-urban migrants (23.98%); education through high school (31.99%) or above is much higher than that of migrants (12.31%). More rural-to-urban migrants (20.11%) did not have medical insurance than urban residents (15.26%). Rural-to-urban migrants had a higher proportion of being employed than did urban residents. Although migrants had a higher income, they were less satisfied with their income than were residents. Simultaneously, migrants reported a lower self-assessment of their own social status (4.00 ± 1.78). Migrants had a higher proportion of better self-reported health (66.30%) and a lower proportion of self-reported illness (5.82%) than did urban residents (62.46% and 7.61%, respectively). They had no differences in smoking and drinking. The largest proportion of migrants was found to be in the eastern region (59.95), while the smallest was in the western region (15.01%) of China.

**Table 2:** Distribution difference of characteristics between urban residents and rural-to-urban migrants

	Urban residents (*n* = 11,300)		Rural-to-urban migrants (*n* = 2819)			
	N or M ± SD or P_50_[P_25_, P_75_]	%	N or M ± SD or P_50_[P_25_, P_75_]	%	*F/X* ^*2*^	*P*
Gender					0.0048	0.95
Female	6180	50.26	1569	51.54		
Male	5452	49.74	1379	48.46		
Age	43.7 ± 13.75		36.71 ± 12.18			< 0.001^**^
15~ 24	1278	11.31	463	16.42		
25~ 34	1736	15.36	900	31.93		
35~ 44	2461	21.78	688	24.41		
45~ 59	4281	37.88	641	22.74		
60~	1544	13.66	127	4.51		
Marital status					68.96	< 0.001^**^
Unmarried	1909	16.89	554	19.65		
Married, living together	8808	77.95	2216	78.61		
divorce, widowed	583	5.16	49	1.74		
Education Level					687.30	< 0.001^**^
Primary or less	1099	9.73	676	23.98		
Junior middle school	6586	58.28	1796	63.71		
High school or above	3615	31.99	347	12.31		
Medical insurance					39.15	< 0.001^**^
Yes	9576	84.74	2252	79.89		
No	1724	15.26	567	20.11		
Job					442.62	< 0.001^**^
Yes	5914	52.34	2094	74.28		
No	5386	47.66	725	25.72		
Income in the past year	20,000 [4,45,000]		24,000 [6,42,000]		2.376	0.018^*^
Satisfied with income					40.67	< 0.001^**^
Satisfactory	2984	26.41	610	21.64		
Fair	3699	32.73	1078	38.24		
Unsatisfactory	4617	40.86	1131	40.12		
Self-assessment of social status	4.57 ± 1.80		4.00 ± 1.78		227.33	< 0.001^**^
Self-reported health					18.46	< 0.001^**^
Health	7058	62.46	1869	66.30		
Fair	3382	29.93	786	27.88		
Unwell	860	7.61	164	5.82		
Survey area					279.68	< 0.001^**^
East	4796	42.44	1690	59.95		
Central	3940	34.87	706	25.04		
west	2561	22.69	423	15.01		

** significance *P* < 0.001; * *P* < 0.05

### Needs and utilization of health services

The differences in needs and utilization of health services between urban residents and rural-to-urban migrants are reported in Table 3. Rural-to-urban migrants (11.99%) had higher health services needs than did urban residents (10.47%) in general, while in 2012, urban residents and migrants had no differences. However, there was no difference in the utilization of health services between residents and migrants.

**Table 3:** The difference of needs and utilization of health services between urban residents and rural-to-urban migrants

	Urban residents			Rural-to-urban migrants			*X* ^*2*^	*P*
	Yes	No	Rate (%)	Yes	no	Rate (%)		
Needs	1183	10,117	10.47	338	2481	11.99	5.43	0.020^*^
2012	658	2992	18.03	125	585	17.61	0.072	0.79
2014	249	3957	5.92	108	961	10.10	23.63	< 0.001^**^
2016	276	3168	8.01	105	935	10.10	4.45	0.035^*^
Utilization	710	473	60.02	208	130	61.54	0.25	0.61
2012	423	235	64.29	84	41	67.20	0.39	0.53
2014	152	97	61.04	61	47	56.48	0.65	0.42
2016	135	141	48.91	63	42	60.00	3.75	0.053

** significance *P* < 0.001; * *P* < 0.05

Three logistic models of health services needs are reported in Table 4. In model 1, we included all subjects in the model. Using the urban migrants as a reference group, this revealed in the model of rural-to-urban migrants (OR = 1.19, *P* < 0.05) that health services needs were significantly higher than those of the residents when controlling for other variables. We also separated residents and migrants to build two regression models. For the control variables, age had a negative relation to health services needs in model 1 (OR = 0.99, *P* < 0.001) and model 2 (OR = 0.98, *P* < 0.001). Males had a higher health services needs in all three models (OR = 0.77, *P* < 0.001; OR = 0.78, *P* < 0.001; OR = 0.74, *P* < 0.05). Those who had medical insurance had higher needs in model 1 (OR = 1.34, *P* < 0.001) and 2 (OR = 1.43, *P* < 0.001), but there were no significant influences in model 3. In model 1, those who were dissatisfied with their own income had higher needs than those who were satisfied with their income.

**Table 4:** OR of health services needs between urban residents and rural-to-urban migrants

Variable	Model 1 Total	Model 2 Urban residents	Model 3 Rural-to-urban migrants
	OR(95%CI)	OR(95%CI)	OR(95%CI)
Urban resident (Ref)			
Rural-to-urban migrant	1.19^*^(1.03,1.37)	–	–
Age	0.99^**^(0.98,0.99)	0.98^**^(0.98,0.99)	0.99 (0.98,1.00)
Female (Ref)			
Male	0.77^**^(0.69,0.86)	0.78^**^(0.69,0.89)	0.74^*^(0.58,0.95)
Medical insurance (Ref = no)	1.34^**^(1.13,1.59)	1.43^**^(1.17,1.76)	1.13 (0.83,1.53)
Satisfied with income (Ref)			
Fair satisfied with income	1.12 (0.95,1.32)	1.14 (0.94,1.36)	1.09 (0.76,1.56)
Unsatisfied with income	1.32^**^(1.12,1.54)	1.30^**^(1.09,1.57)	1.33 (0.94,1.89)
Self-report of health (Ref = Health)			
Fair	3.00 (2.63,3.42)	3.10^**^(2.66,3.60)	2.56^**^(1.96,3.35)
Unwell	9.60 (8.05,11.46)	9.51^**^(7.81,11.59)	9.32^**^(6.41,13.77)
Area (Eastern Ref)			
Central	0.82^**^(0.72,0.94)	0.82^**^(0.71,0.95)	0.86 (0.65,1.16)
Western	0.96 (0.83,1.12)	0.98 (0.83,1.16)	0.85 (0.59,1.22)
2012 (Ref)			
2014	0.34^**^(0.30,0.39)	0.31^**^(0.26,0.36)	0.51^**^(0.38,0.68)
2016	0.43^**^(0.37,0.49)	0.41^**^(0.35,0.48)	0.53^**^(0.39,0.71)

** significance *P* < 0.001; * *P* < 0.05

## Discussion

The number of rural-to-urban migrants was large and showed an upward trend in the past few years. This result resembled the analysis of the Migrants Population Dynamic Monitoring Survey Data by National Bureau of Statistics.^5^ It seems that rural-to-urban migrants are still a growing group and shall continue to attract the government’s concerns.

In the study, we determined that rural-to-urban migrants had higher needs. The recent two-week prevalence rate of illness among rural-to-urban migrants was 11.99%. Compared to other studies in China, the outcomes were similar to that of general migrants in Beijing [30], Shanghai and Pearl River Delta Areas of Guangdong [31], with a rate ranging from 11 to 13.1%, but lower than Shenzhen (24.51%) [32] and Xi’an (18.22%) [33]. Most studies directly compared their results with the data of the NHS (National Health Service) Survey, drawing the conclusion that the need of health services for migrants was lower than that of the general population. In contrast, we found rural-to-urban migrants (11.99%) had higher health needs than urban residents (10.47%, *P* < 0.05), and was supported by model 1’s results(OR = 1.19, CI: 1.03–1.37). Besides, more serious illnesses of the migrants might be associated with their poorer living conditions and socioeconomic status (SES) [34]. Thus, we focus on discovering the associated factors of health services need between rural-to-urban migrants and urban residents, searching for a new angle to promote health equality.

First, age and gender showed to have influence on health service needs, which was confirmed in other studies [35]. In models 1 and 2, age was a negative factor of health service needs, but no significant differences in model 3. The rural-to-urban migrants were younger than urban residents, and most of the migrants were under 44 years old, thus weakening the effect of age on health needs. Both female migrants (male’s OR = 0.74, CI: 0.58–0.95) and female residents (male’s OR = 0.78, CI: 0.69–0.89) had higher health needs. One possible reason could be that females presumably were more aware of health problems and symptoms than males [36]. Or it could be that most female migrants moved into the urban setting because of marriage, and it was hard to get used to urban life, thus, they experience more mental or physical health issues.

Second, we determined that urban residents who were dissatisfied with their income had higher health needs (OR = 1.30, CI: 1.09, 1.57), while rural-to-urban migrants did not show a significant difference. Besides, there was no significant difference between personal income and health needs. It turned out that regardless of income level, those who were dissatisfied with their income might have to push themselves to work harder or just complain a lot, either of these would end up in mental health problem if they didn’t learn to deal with stress. Almost all rural-to-urban migrants currently focus on their economic conditions only, ignoring their own health, including physical health and mental health. In addition, with the progress of urbanization and the reform of the household registration system [37], an increasing number of rural-to-urban migrants would become urban residents. Mental health issues may begin to surface. Therefore, we suggest that the government pay attention to mental health and prevent urban residents from suffering mental health.

Third, the health system is the main factor influencing health needs, and medical insurance plays an important part of the health system. We found urban residents with medical insurance had higher health needs, which is in accordance with previous studies [38]. We have two possible reasons. One is that high-quality health insurance requires a high price; it is also possible that those with medical insurance are more willing to invest in their health and have regular health examinations. However, we did not find the same results in the migrant model. Fewer rural-to-urban migrants who had medical insurance (79.89%) than urban residents (84.74%). Although the majority of rural-to-urban migrants are pursuing a better life in the urban areas, they are still fighting for more income and thus neglect their own health. According to the National Development and Reform Commission, at the end of 2011, there were more than 1.3 billion people participating in the basic medical insurance plan for urban and rural residents in China, with a coverage rate of more than 95%.^6^ Both proportions medical insurance for urban residents and rural-to-urban migrants were below this benchmark, which showed a phenomenon that some people already had basic medical insurance, but did not realize their insured status. This revealed that the basic medical insurance system was not being used effectively, even when the government strongly promoted it. We suggest that the government should strengthen health education and promote health insurance, while promoting national health insurance coverage.

Finally, we found that there were no significant difference in health care utilization between rural-to-urban migrants and urban residents. Contradicting previous studies, we do not agree that rural-to-urban migrants had lower health awareness or more barriers in accessing health services [39].In China, the Floating Population Service Center was established in December 2011 with the approval of the Central Committee and officially was launched in April 2014.^7^ One of the main tasks is to provide information for the management of migrant population health and health care services. This means that the Chinese government is aware of the situation of the floating population and has begun to enhance health and fairness.

Among those who had reported illnesses in the past 2 weeks, 61.54% of rural-to-urban migrants and 60.02% of urban residents had clinical visits within the past 2 weeks (*P* = 0.61), which is much higher than that reported in previous studies in China [30–32]. The improvement of health care utilization may be attributed to the promotion and implementation of health care reform in 2009–2011 [40]. One key issue of this reform is tiered medical services. By signing a contract between family physicians and residents [41], these services have demonstrated efficiency in improving health and promoting health service equity [42]. Although members of the floating population who had migrated more than 6 months prior were incorporated into the Family Physician Sign System, some problems still need to be addressed, such as how to deal with the continuity of signing for those who might migrate to many cites and how to strengthen migrants’ health awareness.

### Limitations

The study suffers from several limitations. It could be significant difference of years between urban residents and migrants and that needed more evidence and researches to prove. As Table 3 showed, we recognized a large drop in need for health services from 2012 to 2014 in both urban residents and migrants (from 18.03 to 5.92 in urban residents and from 17.61 to 10.10 in migrants). In 2012, the questions about health service needs were conducted vaguely. And in 2013, the National Health and Family Planning Commission of PRC operated a pilot work on equalization of basic public services for floating population^6^. Therefore, using questionnaire in the pilot work as a reference, our questionnaire was reconstructed. Giving more definitions and standards, it could make sure both interviewer and interviewee understand well. Besides, recall bias may exist in a self-reported study, but this should be minimal due to the limited recall period of 2 weeks; collecting variables is based on a self-reported method and may be measured with errors; our study is cross sectional and thus does not allow such causality to be determined. And, health service utilization was also determined by the accessibility of health services in terms of geography, cultural and administrative barriers in addition to socioeconomic factors and quality issues. It is necessary to consider more associated factors in the future.

## Conclusion

In conclusion, this study has shown that rural-to-urban migrants had higher health care needs and age and female had negative effect, but the same health care utilization compared with urban residents in China. Also, urban residents with medical insurance have higher health needs. Health policies focusing on health equity should pay more attention to the rural-to-urban migrants in shaping China’s health care system reform.

## Abbreviations

CLDS: Chinese Labor Dynamic Survey
PPS: probability proportionate to size sampling
SES: socioeconomic status

## Footnotes

1
www.who.int/migrants/publications/WHO_IOM_UNOHCHR-Publication.pdf?ua=1

2
www.stats.gov.cn/tjsj/zxfb/201804/t20180427_1596389.html

3 FRAMEWORK OF PRIORITIES AND GUIDING PRINCIPLES TO PROMOTE THE HEALTH OF REFUGEES AND MIGRANTS

4 The data can be applied and obtained from the CLDS of Center for Social Survey, Sun Yat-sen University at http://isg.sysu.edu.cn/node/353. More detail messages about this survey also can be learned on the official website mentioned above or the two previously published papers [35, 43].

5
http://www.chinaldrk.org.cn/

^6^ http://www.nhfpc.gov.cn/ldrks/s3578/201312/74b817920c644bb6a7b78a9d18fd3449.shtml

6
http://www.yaic.com.cn/news/show?cid=34&id=1216

7
http://www.chinaldrk.org.cn/wjw/news-update/#notice
